# Diabetes mellitus can cause cardiomyopathy disorders by inducing the aging pathway

**DOI:** 10.22038/ijbms.2021.54783.12287

**Published:** 2021-05

**Authors:** Mahdi Ahmadi, Shirin Saberianpour, Morteza Heidarzadeh, Jafar Rezaie

**Affiliations:** 1 Tuberculosis and Lung Disease Research Center, Tabriz University of Medical Sciences, Tabriz, Iran; 2 Stem Cell Research Center, Tabriz University of Medical Sciences, Tabriz, Iran; 3 Vascular and Endovascular Surgery Research Center, Mashhad Medical University, Mashhad, Iran; 4 Solid Tumor Research Center, Research Institute for Cellular and Molecular Medicine, Urmia University of Medical Sciences, Urmia, Iran

**Keywords:** Aging, Autophagy, Cardiac, Cardiomyopathy, Diabetes mellitus, Signaling pathway

## Abstract

**Objective(s):**

In this study, cardiovascular disorders were examined with a focus on the aging pathway and autophagy involvement in cardiac samples isolated from male rats with type 2 diabetes mellitus.

**Materials and Methods:**

In the present study, male Wistar rats became diabetic with the help of a high-fat diet. Gene and protein expression levels (to evaluate Tumor Necrosis Factor-α, TNF-α) were measured by the ELISA method. Nrf2, p38, and GSK-3β proteins in cardiac tissue samples were measured by the western blotting method. Autophagy examination was performed with immunofluorescence staining. Finally, quantitative results were calculated using statistical analysis.

**Results:**

The expression of beta-galactosidase genes had a significant increase in the diabetic group (*P*=0.0001). However, there was no significant difference in the expression of the SERCA2a gene between the diabetic and control groups. In terms of protein expression, the amount of TNF-α protein in the diabetic group was significantly different from that of the control group (*P*=0.0102). The expression levels of p38, Nrf2, and GSK-3β proteins increased compared with the control group. The use of the LC3 immunofluorescence staining technique revealed that autophagy increased in the diabetic group.

**Conclusion:**

Type 2 diabetes mellitus in rats will increase aging in cardiac cells. Examination of the signaling pathway indicates that this effect is related to the increase of ROS and the activity of the signaling pathway. In response, the cardiac cells try to maintain their homeostasis by increasing autophagy and decreasing inflammatory cytokines.

## Introduction

Cardiovascular complications are some of the most common and deadly clinical consequences of type 2 diabetes (1). The treatment cost of diabetes is very high for countries (2), especially diabetic patients with cardiovascular complications are reported to have lower health levels, lower quality of life, higher rates of depression and disability, and inequality in social and occupational fields (3). Research has shown that the risk factors for cardiovascular complications are complex and multifactorial in type 2 diabetic patients (4). Cardiac cell death with chronic loss of myocytes and vascular structures has been suggested as a major cause of anatomical and functional changes in the diabetic cardiac tissue (5). However, myocyte death and defects in the mechanical behavior of myocytes due to diabetes play a major role in the onset of diabetic cardiomyopathy (6).

Hyperglycemia leads to enzymatic activation, which causes the renin-angiotensin system to become overactive and stimulate angiotensin II (Ang II) synthetization (7). As a result, phosphorylation of p53 by p38 mitogen-activated protein (MAP) kinase chronically stimulates tumor suppression and Ang II formation. Hormone secretion and receptor binding increase both cytosolic Ca^2+^ and production of ROS (8). In this condition, cell death begins, causing DNA damage, telomere shortening, irreversible cessation of growth, and cellular aging. In addition, the number of old myocytes rises considerably and the cardiac tissue becomes weak in contraction and suffers from ventricular failure (9).

Hyperglycemia induces the production of ROS that oxidize cysteine sequences in the Keap1 protein, which destroys the interaction of Keap1 and Nrf2, leading to the accumulation of Nrf2. Nrf2 causes the formation of heterodimers with small proteins such as MAF, which binds to the promoter sequences in the respective genes and causes a negative regulator in the regeneration and dysfunction of the cardiac tissue (10).

Aging is an irreversible biological process caused by a variety of stimuli (11), including high blood sugar and the production of ROS, which is considered an independent risk factor for cardiovascular diseases (12). Aging is associated with increased expression levels of aging-related genes and proteins, including β-galactosidase and SERCA2 (13). The induction of autophagy during aging reduces the effects of this process and in fact can reverse age-related cardiac hypertrophy and contractile dysfunction (14).

Another reaction of the body to maintain cardiac function is the SERCA2a calcium pump’s role in increasing the entry of calcium into the myoplasm to maintain the contractile power of cardiomyocytes (15, 16).

The level of the SERCA2a calcium pump activity is reported to be directly related to the rate of cardiac hypertrophy and cardiac contractions (17). In this study, cardiac disorders were examined with a focus on the aging pathway and autophagy involvement in cardiac tissue samples isolated from male rats with type 2 diabetes mellitus.

## Materials and Methods

***Animal Ethics***

All procedure phases of the current experiment were according to the Care and Use of Laboratory Animals (NIH Publication No.85-23, revised 1996) guideline and approved by the Animal Care Committee of Tabriz University of Medical Sciences (No: TBZMED. REC.1398.243).

***Experimental groups***

Sixteen male Wistar rats (initially weighing 190–210 g) were enrolled in this experiment. All animals were kept in standard cages (4 rats per cage) for 10 days. environmental temperature was maintained at 22±2 °C, with a constant humidity of 45–55%, on a 12-hr light-dark cycle (07.00 on/19.00 off) with free access to food and tap water, before the manipulation. Animals were randomly allocated into two experimental groups (each in 8 rats) as follows: Control groups received regular chow and tap water (C group); Diabetic groups received high-fat diets (HFD; 48% carbohydrate, 22% fat, and 20% protein) and a low dose of STZ (Type 2 diabetes mellitus group).

***Induction of type 2 diabetes mellitus in rats***

Type 2 diabetes Mellitus was induced using HFD feeding for 4 consequent weeks followed by a single intraperitoneal (IP) injection of a low dose of STZ (35 mg/kg; Sigma Aldrich, Germany). After 3 days of STZ injection, a blood sample of the tail vein was obtained and non-fasting plasma glucose was measured using a digital glucometer (Norditalia Elettro medica liSrL., Italy). Rats with the non-fasting plasma glucose ≥300 mg/dl were considered as diabetic status. After induction of type 2 diabetes mellitus, rats received HFD for the next 8 weeks. Thereafter, all animals were euthanized 8 weeks after diabetes confirmation (for molecular and histological analysis of lung tissues). Control rats were given regular food and water throughout the experiments.

***Measuring expression of B*** ***-*** ***galactosidase and SERC2A genes by real-time PCR***

Cardiac tissues after extraction of rats were incubated in -80 °C until use. Prepared tissues were powdered by fluid nitrogen. Afterward, RNA isolation was done using an RNA extraction kit (Cat no: YT9065; YTA Co.). RNA integrity was confirmed by the NanoDrop system (Thermos Scientific NanoDrop™1000). The reverse transcriptase extends a primer complementary to the RNA to produce complementary DNA (cDNA) for the RNA template (Bioneer). We used Oligo 7 software (Molecular Biology Insights Inc.) for primer design. For real-time RT-PCR, cDNA, primer pairs, and SYBR Green dye were used in TaqMan Master Mix (Applied Biosystems). The total mix was run on a Real-Time System (Applied Biosystems by Life Technologies, Carlsbad, CA, USA) with the PCR conditions as follows: 94 °C (30 sec), 59 °C (30 sec), and 72 °C (45 sec) for 40 cycles. The data are presented as the relative mRNA level normalized to GAPDH and then expressed as the fold increase relative to the control.

***Immunofluorescence***

At first, the cardiac tissue isolated from the rats was cut and laminated. The specimens were kept at -20 °C until staining. LC3 immunofluorescence staining was done on slides. Tissues were fixed with methanol (Merck) for 10 min and blocked with 1% BSA (Sigma) then solved in 0.1% Triton X-100 for 1 hr. The tissues were incubated with the anti-rat LC3 primary antibody (1:200) (Cat no: ab51520) at room temperature for 1 hr and washed three times with PBS. Samples were viewed and photographed with upright microscopy (Model: BX51, Olympus) and the images were processed by Cell Sense Software Ver.1.4.

***Level of TNF-*** ***α***

Protein extraction of cardiac tissue was performed. Cardiac tissue was homogenized using liquid nitrogen in a Chinese mortar. It was incubated with ice-cold protein lysis buffer (NaCl, Tris-HCl, NP-40 supplemented with inhibitor) for 30 min. The soluble protein samples were centrifuged at 11,000 rpm for 20 min and supernatant harvested and stored at -20 °C until use. TNF-α was assessed using the TNF-α Enzyme-Linked Immunosorbent Assay (ELISA) test (BioSource International, Human TNF-α, Belgium). The BioSource International TNF-α kit is a solid phase sandwich ELISA method in which the antigen is conjugated with the specific antibody for TNF-α in the first incubation and the biotin-coated antibody is added after washing. In the second incubation, this antibody was bound to the TNF-α that was detected in the first incubation. After the removal of the excess secondary antibody, streptavidin-peroxidase was added. The four-layered sandwich was completed after the biotin-coated antibiotic was bound. Substrate solution was added after the third incubation and removal of the unbound enzymes. The color was obtained after it was bound to the enzyme. The density of the colored product was directly proportional to the concentration of TNF-α. Then absorbance levels were read at 450 nanometer wavelength and patient samples were evaluated.

***Evaluating the protein content of GSK3B, Nrf2, and P38 by Western blotting***

Protein was isolated from cardiac tissue, 50 μg of protein lysate was loaded in each lane of 10% sulfate-polyacrylamide gel electrophoresis (Bio-Rad Laboratories, Inc., Hercules, CA, USA) and transferred to nitrocellulose membranes (Pierce). The membranes were blocked for 2 hr in a non-fat dried milk solution (5% in Tris-buffered saline) containing 0.5% Tween 20 and then incubated with proprietary primary antibodies for 2 hr at 4 °C. Specific protein expressions were revealed by enhanced chemiluminescent reagents (Thermo Scientific, Beijing, China) and detected using X-ray film. GAPDH was used as loading control. The data were presented as fold increases relative to the control.

## Results

***The expression level of*** ***the β-galactosidase gene was increased in the diabetic group***

**Figure 1:**
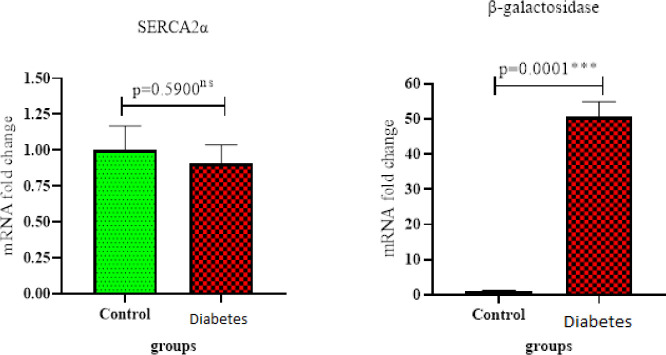
Real-time PCR analysis of β-galactosidase gene in rats with diabetes mellitus group compared with the control group. Data showed that the transcription level of SERiCA2a gene has no significant meaning by statistical analysis in rats with diabetes mellitus group (*P*=0.5900), (N=3)

Monitoring the levels of SERCA2a and β-galactosidase genes showed an increase in expression of β-galactosidase in rats with diabetes mellitus group (*P*=0.0001(N=3)) (Figure1). Real-time PCR analysis showed no significant difference for the SERCA2a gene between the two groups. Data showed that the transcription level of SERiCA2a gene has no significant statistical analysis difference in rats with diabetes mellitus group (*P*=0.5900, (N=3).

***Level of expression proteins GSK3B, Nrf2, and P38 increased in*** ***rats with diabetes mellitus*** ***group ***

**Figure2:**
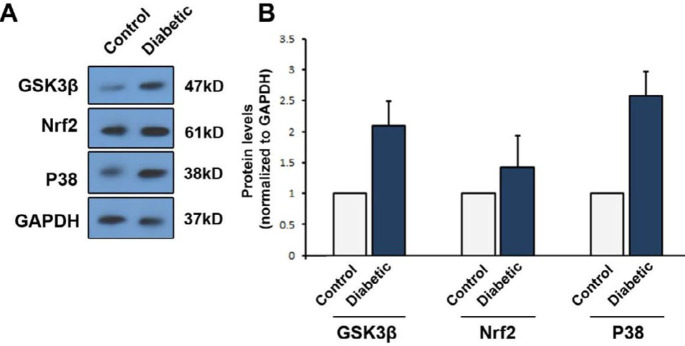
Levels of GSK3B, Nrf2, and P38 detected by Western blotting. Data showed that GSK3B, Nrf2, and P38 contents were increased after induction of diabetes mellitus (N=3)

Western blot showed an increase in the protein levels of GSK3B, Nrf2, and P38 in rats with diabetes mellitus in comparison with the control group (Figure 2). Increasing of expression P38 was almost 2.5 fold compared with control, and the amount for Gsk3B arrived at 2 fold and for Nrf2 0.5 fold compared with the control group.

***Level of TNF- α decreased after induction of diabetes mellitus in rats***

**Figure 3:**
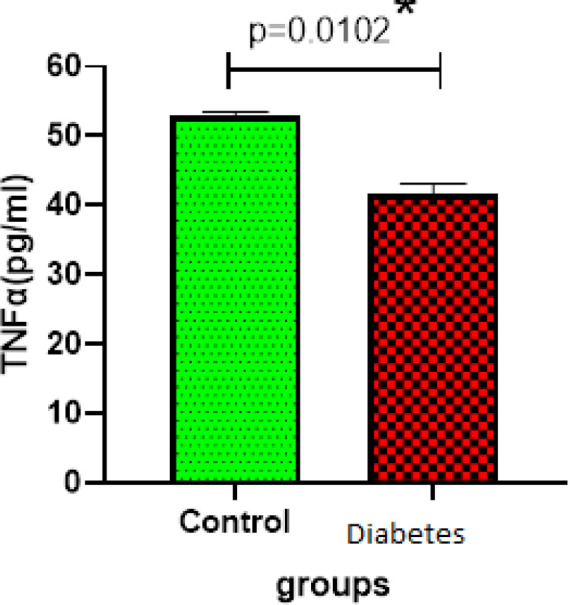
Measuring the level TNF-a ELISA(n=3) in control and diabetes groups. Data showed decreased TNF-α after Induction of diabetes mellitus (*P*=0.0102

TNF-α plays a key role in the cellular responses to inflammation and injury. The level of TNF- α was decreased with significant differences in statistical analysis between control and Diabetes groups (*P*=0.0102) (Figure 3).

***Autophagy increased after Induction of diabetes mellitus in rats***

**Figure 4:**
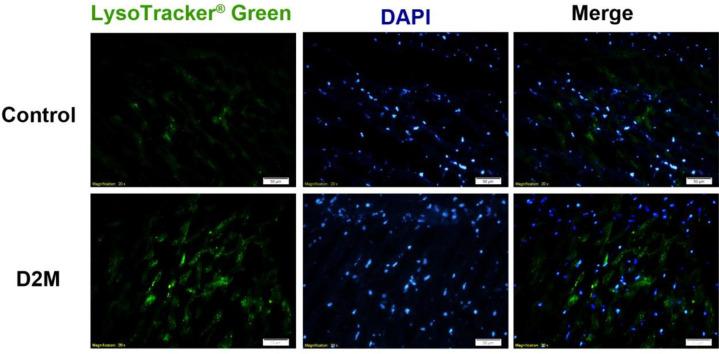
Immunofluorescence images of rat cardiac tissue before and after induction of diabetes type 2

Autophagy is a regulator for the destruction and clearance of organs and proteins of cell damage. In this process, the autophagous cells, by combining with lysosomes, produce the enzymes needed for this cell cleansing. This process can be done by immunohistochemistry staining. Cells were stained with LysoTracker Green B, counterstained for nuclei with DAPI (blue) and C, merge of two pictures for better comparison. The graph indicates increased autophagy in the diabetic group in comparison with the control group. The results show an increase in phagolysosomes in the diabetic group compared with the control group in Figure 4.

## Discussion

The most common cause of death and morbidity in diabetic patients is cardiovascular complications, among which cardiomyopathy is very common in people with type 2 diabetes. According to the results of the present study, the expression of β-galactosidase genes had a significant increase in the diabetic group (*P*=0.0001). However, there was no significant difference in the expression of the SERCA2a gene between the diabetic and control groups. In terms of protein expression, the amount of TNF-α protein in the diabetic group was significantly different from the control group (*P*=0.0102).

The expression of p38, Nrf2, and GSK-3β proteins increased in the diabetic group compared with the control group. Immunofluorescence staining revealed that autophagy increased in the diabetic group. The results showed increases in proteins and genes associated with aging. Moreover, elevated expression levels of Nrf2, GSK-3β, and p38 proteins were also observed due to high concentration of glucose and the increase of ROS signaling of the associated signaling pathway. Increased accumulation of Nrf2 protein in combination with other proteins increases the expression of aging-related genes such as β-galactosidase.

As shown in previous studies, diabetes mellitus leads to decreased cardiac function and structural degeneration in the primary aging process, but it can become secondary due to other disorders (18). Fomison found that increased glucose levels induced cellular aging by increasing miR-34a (19). Another study by Ryder *et al*. showed that obesity and type 2 diabetes increased both systolic blood pressure and vascular aging (20). Aging characterizes a wide range of injuries with gradual loss of tissue and/or function of cardiac cells, including fibrosis, hypertrophy, overgrowth of the cardiac muscle, and cardiomyopathy (21).

On the other hand, age-related processes, such as inflammation, autophagy, and mitochondrial dysfunction, are interconnected biological processes (22). Increased autophagy plays an important role in maintaining cardiac function against aging (23). Increased autophagy and the inhibition of aging-induced apoptotic cells delay the effects of cellular aging such as hypertrophy and cardiac fibrosis (24). Along with increased expression levels of aging-related genes and proteins in our diabetic rats, autophagy also increased in this diabetic group compared with the control group (24). A study discovered the TGFB-INHB/activing-mediated inhibition of TORC2 as a novel mechanism for age-dependent decreases in autophagy activity and cardiac health (25). This mechanism applies to other tissues of the body besides the cardiac tissue (26).

In this regard, Cao *et al.* presented evidence that regulation of autophagy through the Akt/FoxO3a signaling pathway reduced the effects of aging in liver cells (27). TNF-α is a proinflammatory factor whose normal expression can contribute to tissue function. Excessive expression of this cytokine causes cardiac dysfunction, especially left ventricular (LV) dysfunction (28, 29). In the present study, the level of TNF-α decreased compared with the control group. TNF-α cytokine production may play a protective role in cardiac adaptation after injury or environmental stress.

Thus, the role of TNF-α in myocardial homeostasis can be constructive. The data indicates that type 2 diabetes mellitus in rats will increase aging in cardiac cells. By examining the signaling pathway, this effect is related to the increase of ROS compounds and the activity of the signaling pathway. In response, the cardiac tissue tries to maintain its homeostasis by increasing autophagy and decreasing inflammatory cytokines.

**Table1:** Primer list for RT-PCR

Sequences(5´-3´)	Gene
F: GAT GGG TGT GAA CCA CGA R: ACG GAT ACA TTG GGG GTA	GAPDH
F: CAAATCCCACCTTGAACACA R: CGACTGACTAATGGCAGCAG	Nrf2
F: CTACACCAA CGTAACCTATCCC R: TTCTCCGGCGCGTAAAAATGC	β- galactosidase

## Conclusion

Type 2 diabetes mellitus in rats will increase aging in cardiac cells. Examination of the signaling pathway indicates that this effect is related to the increase of ROS and the activity of the signaling pathway. In response, the cardiac cells try to maintain their homeostasis by increasing autophagy and decreasing inflammatory cytokines.

## Conflicts of Interest

The authors declare that no conﬂicts of interest exist.
